# Regional Anesthetic and Analgesic Techniques for Clavicle Fractures and Clavicle Surgeries: Part 2—A Retrospective Study

**DOI:** 10.3390/healthcare10101987

**Published:** 2022-10-10

**Authors:** Chang Chuan Melvin Lee, Chong Boon Lua, Kailing Peng, Zhi Yuen Beh, Shahridan Mohd Fathil, Jin-De Hou, Jui-An Lin

**Affiliations:** 1 Department of Anesthesia, Toowoomba Base Hospital, Darling Downs Health, Toowoomba City, QLD 4350, Australia; 2 Rural Clinical School, Toowoomba Regional Clinical Unit, University of Queensland, South Toowoomba, QLD 4350, Australia; 3 Department of Anesthesia, National University Health System, Singapore 119074, Singapore; 4 Center for Regional Anesthesia and Pain Medicine, Chung Shan Medical University Hospital, Taichung 40201, Taiwan; 5 OSC Orthopaedic Specialist Centre, Subang Jaya 47600, Selangor, Malaysia; 6 Department of Anesthesiology, Assunta Hospital, Petaling Jaya 46990, Selangor, Malaysia; 7 Department of Anesthesiology, Gleneagles Hospital Medini Johor, Iskandar Puteri 79250, Johor, Malaysia; 8 Center for Regional Anesthesia and Pain Medicine, Wan Fang Hospital, Taipei Medical University, Taipei 116, Taiwan; 9 Division of Anesthesiology, Hualien Armed Forces General Hospital, Hualien 97144, Taiwan; 10 Department of Anesthesiology, School of Medicine, National Defense Medical Center, Taipei 11490, Taiwan; 11 Department of Anesthesiology, Wan Fang Hospital, Taipei Medical University, Taipei 116, Taiwan; 12 Department of Anesthesiology, School of Medicine, College of Medicine, Taipei Medical University, Taipei 110, Taiwan; 13 Pain Research Center, Wan Fang Hospital, Taipei Medical University, Taipei 116, Taiwan; 14 Department of Anesthesiology, School of Medicine, Chung Shan Medical University, Taichung 40201, Taiwan; 15 Department of Anesthesiology, Chung Shan Medical University Hospital, Taichung 40201, Taiwan

**Keywords:** analgesia, anesthesia, clavicle, fractures, bone, motor activity: motor-sparing, nerve block, pain, postoperative

## Abstract

**Objective.** Clavicle fracture fixation is commonly performed under general anesthesia due to the complex sensory innervation in this region which poses a challenge for anesthesiologists applying regional anesthetic (RA) techniques. In part 1 of this two-part study, we summarized the current literature describing various RA approaches in clavicle fractures and surgery. In our earlier scoping review, we surmised that a superficial or intermediate cervical plexus block (CPB) may provide analgesia for this procedure and, when combined with an interscalene brachial plexus block (ISB), can provide anesthesia to the clavicular region for surgical fixation. We performed a retrospective study, consolidating assumptions that were based on the results of our earlier scoping review. **Methods.** A retrospective study was conducted on 168 consecutive patients who underwent clavicle fixation surgery at a tertiary healthcare system in Singapore. We used a standardized *pro forma* to collate perioperative data from the electronic health records of both hospitals, including anesthetic technique, analgesic requirements, pain scores, and adverse events, up to the second postoperative day or up until discharge. **Results.** In our study, patients who received RA had significantly reduced pain scores and opioid requirements, compared to general anesthesia (GA) alone. Through subgroup analysis, differences were found in postoperative pain scores and opioid requirements in the following order: GA alone > GA with local infiltration analgesia > CPB > CPB plus ISB. All patients who received combined CPB and ISB had upper limb weakness in recovery, compared to none with CPB alone (*p* < 0.001). Of those who received an ISB either in isolation or combined with a CPB, four (9.3%) were reported to have dyspnea (within 24 h) and motor weakness that persisted beyond 12 h, compared to none for patients that received CPB alone. **Conclusions.** Addition of a CPB to GA for clavicle fracture fixation surgery is associated with reduced pain scores in the early postoperative period, with a lower opioid requirement compared to GA alone. In patients undergoing GA, the combination of a CPB with an ISB was associated with a small, although statistically significant, reduction in pain scores and opioid requirements compared to a CPB alone.

## 1. Background

Clavicle fractures are a common upper extremity injury that can be associated with significant perioperative pain [1,2,3]. Surgical fixation is traditionally performed under general anesthesia (GA) due to the complex, overlapping sensory supply in this region, coupled with limited experience with regional anesthesia (RA) in this patient group [4,5,6,7]. The exact sensory innervation to the clavicle region is contentious, and remains a subject of ongoing debate [5,6,7]. In part 1 of this two-part publication, we articulated that, despite the challenges, clavicle fixation surgery performed under RA, with or without sedation, is feasible, and has been previously reported. We summarized the different combinations of peripheral nerve blocks targeting the cervical plexus, brachial plexus, or their branches that are described in the literature, as well as novel fascial plane approaches [4,8]. In our earlier scoping review, we surmised that the preferred RA technique at present involves a superficial or intermediate cervical plexus block (CPB), combined with an interscalene brachial plexus block (ISB), if complete anesthetic or analgesic cover is required [4,9,10,11,12]. However, an ISB is associated with a number of problems, chiefly amongst which is the propensity to result in hemidiaphragmatic paresis, due to its close proximity to the phrenic nerve. Such a blockade may lead to respiratory compromise in vulnerable patients [4]. This is compounded by sensorimotor blockade of the brachial plexus, which can adversely affect early return‐to‐function and recovery, and impede neurovascular function postoperatively [13]. Considering these risks, it is necessary to determine the magnitude of additional analgesic benefit that is obtained by performing an ISB, particularly in patients who ultimately receive GA. 

Given the lack of studies that directly investigate the impact of a CPB in patients who receive clavicle surgery under GA, and to make our earlier scoping review more complete, we thus conducted a retrospective medical records study in order to consolidate the assumptions based on the findings in our scoping review. Furthermore, in our earlier scoping review, we also noted that the effect of surgical local anesthetic (LA) infiltration as a supplement to patients undergoing clavicle fracture fixation under GA has not been well articulated. In our study, we retrospectively compared patients who had a CPB or ISB alone or in combination, compared to patients who did not receive any regional anesthesia/analgesia technique in the case of clavicle surgery under GA. We also made comparisons between patients who received GA alone versus those who received GA with local infiltration analgesia (LIA) without regional anesthesia (RA).

## 2. Materials and Methods

We conducted a retrospective review of patients who underwent clavicle fixation surgery within a tertiary healthcare cluster in Singapore. Approval for the study was obtained from the institutional review board prior to commencement of the study (reference number: DSRB 2021/00596). 

**Data collection.** Data were extracted from the electronic health records of 176 consecutive adult patients undergoing clavicle fixation surgery at 2 hospitals within a tertiary healthcare system, from August 2015 to April 2021. Data were recorded using a standardized pro forma, and included demographic data, such as age, gender, and ethnicity, relevant anesthetic and surgical data such as anesthetic technique (RA alone, GA alone, or combination of both), postoperative pain scores in recovery, opioid requirements (intraoperative and in recovery), non-opioid analgesic regimen, and pain scores up to the 2nd postoperative day, or up to discharge. Documentation from medical, nursing, anesthetic, acute pain services, and surgical records, as well as electronic medication administration charts, were all examined.

Pain scores were expressed as a numeric rating scale from 0–10/10. We reported total opioid requirements from the intraoperative period up to the point of discharge from recovery, as oral opioids are not typically prescribed in recovery at our institution. Total opioid requirements were expressed as milligrams of oral morphine equivalents (OME). We also collected data on block-related complications, and opioid-related adverse effects. Data were manually collated and cross-checked by 2 individuals to ensure accuracy.

**Inclusion and exclusion criteria.** All patients aged 18 years and above who underwent isolated clavicle fixation surgery were considered eligible for inclusion, regardless of the site of the fracture. We excluded 12 patients who either (1) underwent concurrent surgical procedures, or (2) had concomitant non-clavicular injuries due to the potential for distracting pain that would confound the pain assessment. A total of 170 patients were eligible for inclusion into the study.

**Statistical analysis.** Analyses were performed using R statistical software (version 4.1.0; R Foundation for Statistical Computing, Vienna, Austria) and R studio (version 1.4.1717; Rstudio, PBC, Boston, MA, USA). The Kolmogorov–Smirnov test was applied to assess the distribution of continuous variables for normality. Continuous, normally distributed data were expressed in terms of the mean and standard deviation. Pain scores were presented in terms of median and interquartile range. Categorical data were described in terms of numbers and proportions. When multiple hypotheses are tested simultaneously, there is a propensity for type I error, and methods to control the family-wise error rate or false discovery rate are required [14]. When performing comparisons across multiple groups, we used a one-way analysis of variance (ANOVA), adjusting the *p*-value using a Bonferroni correction for quantitative, normally distributed data. In the case of non-parametric data, we applied the Kruskal–Wallis test across groups, and performed post hoc pairwise comparisons using the Wilcoxon rank sum test, controlling the family-wise error rate with Holm’s step-down procedure for multiple testing correction. For statistical significance, we used a threshold of *p* < 0.05.

## 3. Results 

**Patient characteristics.** Patient characteristics are presented in Table 1. There were no statistically significant differences in age, gender, or ethnicity between patients who underwent GA alone compared to those who received a block. (Table 1). There was no statistically significant difference in fracture site (medial, middle, or distal thirds) in patients who underwent surgery under GA alone, compared to those who received RA (*p* = 0.186) (Table 1).

In our data set, 87 patients underwent fracture fixation under GA alone. Most patients who received general anesthesia, with or without a block, were intubated (70.5%); there were no differences in the type of airway device used (intubation vs. supraglottic airway) between the group that received regional anesthesia and the group that did not (*p* = 0.648). Amongst patients who received RA, 38 (46.9%) patients received a CPB, 32 (39.5%) patients received a combination of a CPB and an ISB, and 11 (13.6%) patients received an ISB alone. A further two patients received a supraclavicular brachial plexus block (Table 1). Given the small numbers of patients, we excluded the two patients who received a supraclavicular brachial plexus block. Thus, a total of 168 patients were included in the final analysis—81 patients in the group that received RA, and 87 patients in the group that did not receive RA. Of the patients who did not receive RA, 57 (65.5%) received LIA.

**Regional anesthesia vs. general anesthesia.** We first compared patients who received a block versus those who underwent clavicle fixation under GA alone. Varying concentrations (ropivacaine 0.4–0.75%, and bupivacaine 0.25–0.5%) and volumes (CPB mean volume 10.6 ± 4.3 mL; ISB mean volume 10.5 ± 4.1 mL) of LA have all been used at the discretion of the primary anesthetic team. No additives such as dexamethasone, clonidine, or dexmedetomidine were added into any of the nerve block injectates. We found that patients who received RA had a small reduction in median pain scores in the post-anesthetic care unit (1.0 vs. 4.0, *p* < 0.001), and on the first postoperative day (2.0 vs. 4.0, *p* < 0.001) compared to those who did not receive a regional block (Table 2). This was coupled with a reduction in opioid requirements intraoperatively and in recovery (mean OME dose 9.1 mg vs. 28.8 mg, *p* < 0.001), as well as a need for rescue analgesia in recovery (22.2% vs. 50.6%, *p* = 0.002). There were no differences in the incidence of pain that affected sleep (1.1% vs. 3.7%, *p* = 0.580). We did not analyze postoperative opioid use in our institution, as patients are generally not prescribed strong opioids for this procedure postoperatively, and only six patients were administered morphine or oxycodone in the early postoperative period after leaving the operating theatre, hence precluding any meaningful statistical comparison. We also found a statistically significant reduction in postoperative nausea and vomiting in the group that received RA (6.2% vs. 18.4%, *p* = 0.045). There were no reports of oversedation or acute urinary retention that required urinary catheterization.

**Cervical plexus block vs. combined cervical plexus and interscalene block.** We also compared patients who received a cervical plexus block against those who received a cervical plexus block and ISB combination (Table 3). 

During data extraction, we encountered difficulties in nomenclature for cervical plexus blockade, with a near-universal label of superficial cervical plexus block used in clinical documentation. Given the retrospective nature of the study, it is difficult to ascertain if a superficial or intermediate cervical plexus block was performed, particularly in the context of ultrasound guidance; thus, we applied a generic label of CPB for this study. Nonetheless, for the group which received a CPB alone, the landmark technique was associated with a higher opioid requirement (*p* = 0.045) on the basis of linear regression analysis. However, we did not find any statistically significant differences in pain scores (*p* = 0.452, 0.538, and 0.491 for recovery, first postoperative day, and second postoperative day, respectively) in patients who had the block performed under ultrasound guidance compared to the landmark technique.

**Figure 1:**
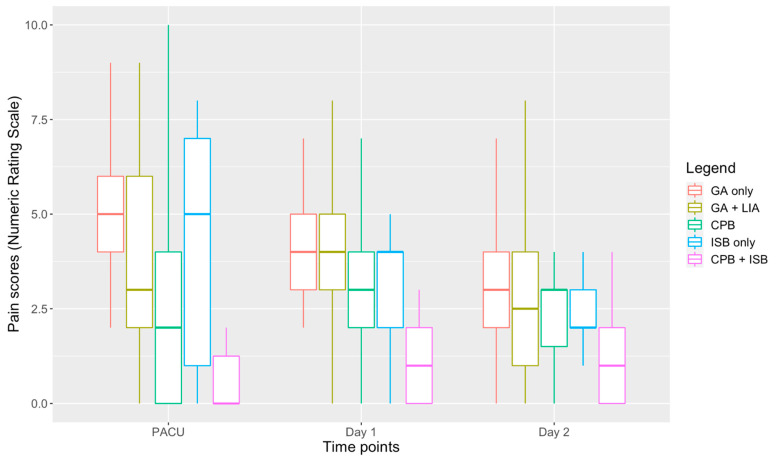
Comparison of pain scores of patients who received general anesthesia alone, compared to those who received different combinations of regional anesthesia. Abbreviations: CPB, cervical plexus block; PACU, post-anesthetic care unit.

**Figure 2:**
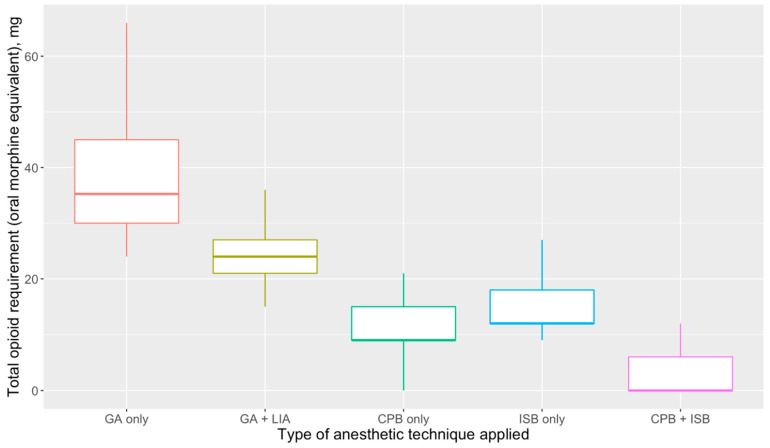
Comparison of total opioid requirement (in terms of oral morphine equivalents) in patients who received general anesthesia alone, compared to those who received different combinations of regional anesthesia in the intraoperative period and post-anesthetic care unit. Abbreviations: IV, intravenous; CPB, cervical plexus block; PACU, post-anesthetic care unit.

In our data set, 80.0% of all CPBs were performed under ultrasound guidance, with no significant difference found between the group that received a CPB alone versus the group that received a combination CPB and ISB (73.7% vs. 87.5%, *p* = 0.150). Compared to patients who only received a CPB, patients who received an ISB in combination with a CPB had lower median pain scores in recovery and on the first postoperative day (0.0 vs. 2.0, *p* = 0.003; and 1.0 vs. 3.0, *p* = 0.019) (Table 3, Figure 1), and lower opioid requirements intraoperatively and in the immediate postoperative period (mean OME dose 4.0 mg vs. 11.1 mg, *p* < 0.001) (Figure 2). Furthermore, three of the patients who received a CPB and an ISB underwent surgery under RA with sedation, which, as with previous studies, demonstrates the feasibility of using the combination as an anesthetic technique where GA is less appropriate or high risk [4,10,12]. In all three cases, patients were administered a target-controlled infusion of propofol, with a peak concentration target that ranged from 1.8–2.5 μg·mL^−1^ with fentanyl 50–100 μg or ketamine 20–40 mg.

**Cervical plexus block vs. general anesthesia with local infiltration analgesia.** In addition, given the lack of studies investigating a CPB and GA compared to GA alone, we compared patients who received a CPB alone to patients who did not receive a RA technique. Furthermore, most studies did not make a comparison between GA with CPB and GA with LIA. In our data set, LIA was typically performed by the surgical team with 10–20 mL of 0.25% or 0.5% bupivacaine with adrenaline. Patients who received a CPB had a small but significant reduction in pain scores during recovery (2.0 vs. 3.0, *p* = 0.009) that was achieved with lower opioid requirements (mean OME dose 11.1 mg vs. 23.9 mg, *p* < 0.001), and a lower need for rescue analgesia (18.1% vs. 40.4%, *p* = 0.042) (Table 4, Figure 1 and Figure 2). 

**General anesthesia vs. general anesthesia with local infiltration analgesia.** We also performed comparisons between patients who received GA alone and those who received GA with LIA. We found that LIA was associated with improved pain scores in recovery (2.0 vs. 3.0, *p* = 0.009), which was achieved with lower opioid requirements (mean OME dose 23.9 mg vs. 38.2 mg, *p* < 0.001) (Table 5, Figure 1 and Figure 2) and a reduced need for rescue analgesia in recovery (40.4% vs. 63.3%, *p* = 0.006). However, there were no statistically significant differences in pain scores on the first (*p* = 0.942) or second (*p* = 0.810) postoperative day.

**Interscalene brachial plexus block.** Only a small number of patients received an isolated brachial plexus block (*n* = 11), which made it difficult to carry out meaningful comparative analyses. Median pain scores were 5.0 (IQR = 6.0), 2.0 (IQR = 1.0) and 2.0 (IQR = 1.0) in the post-anesthetic care unit on the first and second postoperative days, respectively. Mean oral opioid requirements (OME) were 15.4 mg, with a wide 95% confidence interval from 9.8 mg to 21.0 mg. Nonetheless, linear regression analysis showed that a fracture involving the middle third of the clavicle was significantly associated with higher total opioid requirements (*p* = 0.001) compared to lateral third fractures, although this was not statistically significant for pain scores in recovery, or on the first or second postoperative days. 

**Block-related complications.** In our data set, all patients who received an ISB were documented to have upper limb motor weakness in recovery or on arrival to the ward, with four (9.3% of all patients who received an ISB) patients having motor blockade that persisted beyond 12 h, postoperatively. None of the patients who received a CPB alone had documented motor blockade. Lastly, four (9.3%) of all patients who received an ISB were documented to experience dyspnea postoperatively within 24 h of the block; this may have been related to phrenic nerve involvement, although none required oxygen supplementation postoperatively beyond the post-anesthetic care unit. All patients who received an ISB (43 patients, 100.0%) had postoperative motor blockade in recovery. As this was a retrospective study, the exact time or duration of resolution of the motor block could not be ascertained. No patients had a residual upper limb motor or sensory blockade that persisted beyond the first postoperative day.

No other recorded block-related complications were found during our retrospective data collection. We did not find any differences in the incidence of postoperative nausea and vomiting or pain affecting sleep between patients who received RA and those who did not. However, our study was not powered to identify these differences. Lastly, due to the limits of retrospective data collection in terms of electronic health records, we were unable to collect sufficient quantitative or qualitative data on motor blockade or block duration for meaningful analysis. 

## 4. Discussion

We conducted this retrospective study to elucidate the efficacy of CPB with or without the addition of an ISB in the early postoperative period, compared to patients who underwent surgery without regional anesthesia. We also made pairwise comparisons between these groups and with patients who received general anesthesia, with or without LIA. 

**General anesthesia versus regional anesthesia.** Our findings demonstrate that early postoperative pain scores and opioid requirements ranked in the following order: GA alone > GA with LIA > CPB > CPB plus ISB. However, this difference became less significant beyond the first postoperative day (Figure 1). Regional anesthesia use was also associated with a lower incidence of postoperative nausea and vomiting (6.2% vs. 18.4%, *p* = 0.045), likely due to reduced opioid use. Compared to patients who underwent clavicle fixation under GA with LIA, patients who received a CPB had a small but statistically significant reduction in pain scores (2.0 vs. 3.0, *p* = 0.032) in the immediate postoperative period, which was achieved by significantly lower opioid doses (mean OME dose 11.1 mg vs. 23.9 mg, *p* < 0.001) and reduced requirements for rescue analgesia during recovery (Table 2, Table 4, Figure 2). Based on our data, there is the suggestion that an ultrasound-guided CPB is more efficacious than the landmark approach in terms of analgesia provision, due to its association with reduced opioid requirements (*p* = 0.045), although we did not find any significant differences in pain scores. This has to be interpreted on the basis of nomenclature difficulties, similarly to those that were highlighted in our earlier scoping review. In our data set, virtually all CPBs were labelled as a superficial cervical plexus block; however, it is possible that some of these were actually intermediate cervical plexus blocks, with deposition of LA beneath the theoretical fascial barrier that may have dichotomized the two blocks by preventing deeper LA spread—although studies have not consistently demonstrated any difference in efficacy between the two [15,16,17]. 

**Superficial cervical plexus plus interscalene block.** There was a statistically significant, but small further reduction in pain scores and opioid requirements with the addition of an ISB (Table 3). In addition, within the small subgroup that received only an ISB, fractures that involved the distal third appeared to be associated with lower total opioid requirements (*p* = 0.001). This was congruent with the sensory blockade produced by this technique, since the subscapular, lateral pectoral and axillary nerves that innervate the lateral aspect of the clavicle receive contributions from the fifth to seventh cervical nerve roots, which were targeted in this approach [7]. We opine that the clinical difference is probably too small to justify routine addition of an ISB to a CPB, particularly in patients who ultimately receive GA as part of the broader anesthetic plan. Even for fractures that involve the lateral third of the clavicle, the potential benefit obtained from an ISB is unlikely to outweigh its risks. However, a CPB alone is inadequate for anesthesia provision, and needs to be combined with GA; if high-risk GA is to be avoided, a combination of a CPB and an ISB is required.

Unsurprisingly, all patients who received a combined CPB and ISB had limb weakness in recovery, compared to none with CPB alone (*p* < 0.001), in a proportion of patients who received an ISB with a blockade persisting beyond 12 h. This could be undesirable for patients who are candidates for ambulatory surgery, as the insensate upper limb may prolong a return to function. Our retrospective data are limited in terms of describing the extent of motor weakness, and no single method was used to quantify the degree of motor blockade; this is an issue that warrants consideration for future studies. Early studies associated the incidence of phrenic nerve involvement with an ISB to be as high as 100%, albeit with a paresthesia technique [18]. The paradigm shift towards ultrasound-guided RA, facilitating accurate needle placement, low-volume injectate, and extrafascial needle tip placement techniques, have markedly reduced this by as much as 70% [19,20]. Nonetheless, hemidiaphragmatic involvement and resultant respiratory compromise remain as key concerns with ISB. Of those who received an ISB, four (9.3%) were reported to have dyspnea (within 24 h), compared to none of the patients who received CPB alone. In one case, the onset of dyspnea occurred almost immediately following block insertion, prior to induction of GA; for all cases of reported postoperative dyspnea, no other plausible etiology was found, and all patients had received 15–20 mL of LA solution. We postulate that sonographic evaluation of hemidiaphramatic excursion or pulmonary function testing may reveal a higher incidence of hemidiaphragmatic paresis, as clinical effects are usually well-compensated, even with reduced pulmonary mechanics on spirometry, and dyspnea appears late [4,21]. 

The inherent risks associated with the ISB make its recommendation difficult for most patients, particularly if avoidance of motor blockade of the upper limb is desired, given that, unsurprisingly, almost all individuals who receive an ISB will have motor blockade; this was an observation also made by Zhuo et al. [4]. This may be minimized with a more targeted approach, such as a superior trunk or isolated C5/6 block [22,23]. Nonetheless, if avoidance of GA is desired, the combination of a CPB with an ISB is a technique that can provide surgical anesthesia for awake surgery [4,24].

**Local infiltration analgesia.** Subcutaneous and/or subperiosteal infiltration of LA at the surgical site can produce a blockade of local nociceptive fibers [25]. Supplementation with LIA was associated with a small reduction in pain scores in recovery compared to GA alone (3.0 vs. 5.0, *p* = 0.032); considering that this can be achieved with reduced opioid requirements (Table 4), and reduces the need for rescue analgesia in recovery (*p* = 0.006), this simple intervention should be given due consideration whenever possible. However, our data could not be used to examine the efficacy of adding LIA to RA, either pre-emptively or as a rescue procedure, which could be examined in future studies. 

In part 1 of our publication, we discussed the complexity of sensory innervation to the skin over the clavicle, and the clavicle periosteum itself [5,6,7]. In this retrospective study, we sought to validate some of the assumptions that were drawn from our prior scoping review. The results from our retrospective study are congruent with those of studies published earlier, which we summarized in part 1 of this study [4,9,10,11,12,26,27]. 

**Unanswered questions.** In this retrospective study, we noted the frequent use of high-concentration amide LAs in relatively large volumes. This carries a risk of spread to the recurrent laryngeal and phrenic nerve; furthermore, high concentrations of LAs carry an increased potential for neurotoxicity [28]. Thus, the volume and concentration of LA solution needs to be rationalized, particularly when the aim is to provide analgesia rather than surgical anesthesia, especially given that prolonged motor blockade and inadvertent hemidiaphragmatic paresis are major concerns. Our data suggest that a CPB alone affords some motor-sparing potential in the early postoperative period, as long-acting LA in an ISB may produce prolonged motor blockage in some patients. In our earlier scoping review, we noted that there was a large variation in the volumes and choice of LA used in CPBs and ISBs, with similarly high concentrations of amide LA, and volumes as high as 20 mL [4,21]. Further studies could examine the comparability of lower LA concentrations and volumes.

**Study strengths and limitations.** Our study involved a reasonable sample size (*n* = 168) compared to similar studies [22]. However, data collection was performed retrospectively, which limited data collection on variables of interest, such as comprehensive assessment of motor blockade, hemidiaphragmatic excursion, or pulmonary function. Furthermore, complications such as Horner’s syndrome and dysphonia were not captured. Additionally, our sample size was not powered to identify secondary outcomes of interest, such as pain affecting sleep, respiratory depression, or patient satisfaction. Although a propensity score was not developed for comparison between groups, we did not find statistically significant differences in the population characteristics. Larger, well-designed prospective clinical studies are required to corroborate our findings, and examine the aforementioned outcomes with greater resolution. Outcomes of future studies, including more recently described techniques such as the clavipectoral fascial plane block, are eagerly awaited. 

## 5. Conclusions

Addition of a CPB to GA for clavicle fracture fixation surgery is associated with reduced pain scores in the early postoperative period, with a lower opioid requirement compared to GA alone. In patients undergoing GA, the combination of a CPB with an ISB is associated with a small, although statistically significant reduction in pain scores and opioid requirements, compared to a CPB alone. However, the magnitude of the reduction is likely clinically insignificant, especially considering the propensity for motor blockade and hemidiaphragmatic paresis that are associated with an ISB. Further observational and randomized studies are required to determine the efficacy and safety of the various RA techniques.

## Figures and Tables

**Table 1:** Characteristics of patients included in the study.

	Regional Anesthesia	No Regional Anesthesia	*p*-Value
	*n* = 81	*n* = 87	
Age, years (mean, SD)	36.2 (11.9)	38.5 (13.3)	0.237
Gender MaleFemale	71 (87.7%) 10 (12.3%)	69 (79.3%) 18 (20.6%)	0.214
Ethnicity Chinese Malay South Asian Others	27 (33.3%) 26 (32.1%) 14 (17.3%) 14 (17.3%)	37 (42.5%) 20 (23.0%) 21 (24.1%) 9 (10.3%)	0.202
Fracture classification Medial third Middle third Distal third	1 (1.2%) 51 (63.0%) 29 (35.8%)	1 (1.1%) 66 (75.9%) 10 (23.0%)	0.186
General anesthesia Yes No	78 (96.4%) 3 (3.6%)	87 (100.0%) 0 (0.0%)	0.219
Regional technique CPB alone CPB plus ISB ISB alone	38 (46.9%) 32 (39.5%) 11 (13.6%)		

Abbreviations: SD, standard deviation; CPB, cervical plexus block; ISB, interscalene brachial plexus block.

**Table 2:** Pain scores and opioid requirements of patients who received a regional anesthetic technique versus those who received general anesthesia alone.

	Regional Anesthesia	General Anesthesia	*p*-Value
	*n* = 81	*n* = 87	
Pain scores (median, IQR) In PACU POD 1 POD 2	1.0 (4.0) 2.0 (2.5) 2.0 (2.0)	4.0 (3.0) 4.0 (2.0) 3.0 (3.0)	**<0.001** **<0.001** 0.270
Oral morphine equivalents, mg (mean, SD) †	9.1 (8.0)	28.8 (10.1)	**<0.001**
Rescue analgesia in PACU Yes No	18 (22.2%) 63 (77.8%)	44 (50.6%) 43 (49.4%)	**0.002**
Non-opioid analgesia * None Paracetamol only NSAID or COX-2 inhibitor only Paracetamol and NSAID or COX-2 inhibitor	9 (11.1%) 17 (32.1%) 3 (3.7%) 52 (64.2%)	14 (14.9%) 37 (41.3%) 6 (6.9%) 44 (51.8%)	0.370
Postoperative nausea and vomiting Yes No	5 (6.2%) 76 (93.8%)	16 (18.4%) 71 (81.6%)	**0.045**
Pain affecting sleep Yes No	3 (3.7%) 78 (96.3%)	1 (1.1%) 86 (98.9%)	0.580

Abbreviations: CPB, cervical plexus block; ISB, interscalene brachial plexus; IQR, interquartile range; POD, postoperative day; SD, standard deviation; PACU, post-anesthetic care unit; NSAID, non-steroidal anti-inflammatory drugs; COX, cyclooxygenase. † Defined as the total opioid use (in milligrams of oral morphine equivalent) in the intraoperative period and in the post-anesthetic care unit. * Defined as administration within 2 h prior to surgery, intraoperatively, or in recovery.

**Table 3:** Comparison of pain scores and opioid requirements of patients who received a cervical plexus block alone versus those who received a cervical plexus block plus interscalene brachial plexus block.

	GA with CPB	CPB Plus ISB	*p*-Value
	*n* = 38	*n* = 32	
Ultrasound-guided CPB Yes No	28 (73.7%) 10 (26.3%)	28 (87.5%) 4 (12.5%)	0.150
Pain scores (median, IQR) In PACU POD 1 POD 2	2.0 (4.0) 3.0 (2.0) 3.0 (1.5)	0.0 (1.3) 1.0 (2.0) 1.0 (2.0)	**0.001** **0.019** 0.527
Oral morphine equivalents (OME), mg (mean, SD) †	11.1 (8.0)	4.0 (5.47)	**<0.001**
Rescue analgesia in PACU Yes No	7 (18.4%) 31 (81.6%)	4 (12.5%) 28 (87.5%)	0.563
Upper limb motor blockade in recovery Yes No	0 (0.0%) 38 (100.0%)	32 (100.0%) 0 (0.0%)	**<0.001**
Persistent upper limb motor weakness (> 12 h post surgery) Yes No	0 (0.0%) 38 (100.0%)	3 (9.4%) 29 (90.6%)	0.244
Respiratory distress or reported dyspnea (within 24 h of block) * Yes No	0 (0.0%) 38 (100.0%)	3 (9.4%) 29 (90.6%)	0.244

Abbreviations: CPB, cervical plexus block; IQR, interquartile range; POD, postoperative day; SD, standard deviation; PACU, post-anesthetic care unit. † Defined as the total opioid use (in milligrams of oral morphine equivalents) in the intraoperative period, and in the post-anesthetic care unit. * Defined as respiratory distress or reported dyspnea within 24 h of block with no other documented plausible etiology.

**Table 4:** Comparison of pain scores and opioid requirements of patients who received a cervical plexus block alone versus those who received general anesthesia with local infiltration analgesia.

	GA with CPB	GA with LIA	*p*-Value
	*n* = 38	*n* = 57	
Pain scores (median, IQR) In PACU POD 1 POD 2	2.0 (4.0) 3.0 (2.0) 3.0 (1.5)	3.0 (4.0) 4.0 (2.0) 2.5 (3.0)	**0.009** 0.942 0.810
Oral morphine equivalents (OME), mg (mean, SD) †	11.1 (8.0)	23.9 (5.7)	**<0.001**
Rescue analgesia in PACU Yes No	7 (18.4%) 31 (81.6%)	23 (40.4) 32 (59.6)	**0.042**

Abbreviations: CPB, cervical plexus block; GA, general anesthesia; LIA, local infiltration analgesia; IQR, interquartile range; POD, postoperative day; SD, standard deviation; PACU, post-anesthetic care unit. † Defined as the total opioid use (in milligrams of oral morphine equivalents) in the intraoperative period, and in the post-anesthetic care unit.

**Table 5:** Comparison of pain scores and opioid requirements of patients who received a general anesthesia alone versus those who received general anesthesia with local infiltration analgesia.

	GA Alone	GA with LIA	*p*-Value
	*n* = 38	*n* = 38	
Pain scores (median, IQR) In PACU POD 1 POD 2	5.0 (2.0) 4.0 (2.0) 3.0 (2.0)	3.0 (4.0) 4.0 (2.0) 2.5 (3.0)	**0.032** 0.751 0.810
Oral morphine equivalents (OME), mg (mean, SD) †	38.2 (10.3)	23.9 (5.7)	**<0.001**
Rescue analgesia in PACU Yes No	19 (63.3%) 11 (36.7%)	23 (40.4%) 32 (59.6%)	**0.006**

Abbreviations: GA, general anesthesia; LIA, local infiltration analgesia; IQR, interquartile range; POD, postoperative day; SD, standard deviation; PACU, post-anesthetic care unit. † Defined as the total opioid use (in milligrams of oral morphine equivalents) in the intraoperative period, and in the post-anesthetic care unit.

## Data Availability

The data presented in this study are available on request from the corresponding author. The data are not publicly available due to the conditions on data sharing with external parties specified in the Institutional Review Board application.
